# Non-linear relationship between TyG index and the risk of prediabetes in young people: a 5-year retrospective cohort study in Chinese young adults

**DOI:** 10.3389/fendo.2024.1414402

**Published:** 2024-08-16

**Authors:** Jianhui Xiao, Li Zhou, Cheng Luo, Yong Han, Zhenhua Huang

**Affiliations:** ^1^ Department of Geriatrics, Shenzhen Hospital of Integrated Traditional Chinese and Western Medicine, Shenzhen, China; ^2^ Department of Emergency Medicine, the First Affiliated Hospital of Shenzhen University, Shenzhen Second People’s Hospital, Shenzhen, China

**Keywords:** TyG index, relationship, prediabetes, young people, non-linear, Chinese adults

## Abstract

**Objective:**

Given the limited evidence on the relationship between the triglyceride-glucose (TyG) index and the risk of prediabetes among young adults, our study aimed to investigate the potential impact of the TyG index on the future development of prediabetes in young individuals.

**Methods:**

This retrospective cohort study included 125,327 healthy adults aged 20 to 45 years. We utilized Cox proportional hazards regression models, combined with cubic spline functions and smooth curve fitting, to assess the relationship between baseline TyG index and the risk of prediabetes among young adults, exploring its non-linear association. A series of sensitivity analyses and subgroup analyses were conducted to ensure the robustness of our findings.

**Results:**

After adjusting for covariates, the study found a positive correlation between the TyG index and the risk of prediabetes (HR=1.81, 95%CI: 1.54–2.13, p<0.0001). The risk of prediabetes increased progressively across quartiles of the TyG index (Q1 to Q4), with Q4 showing a significantly higher risk compared to Q1 (adjusted HR=2.33, 95% CI=1.72–3.16). Moreover, a non-linear relationship was identified between the TyG index and the risk of prediabetes, with an inflection point at 9.39. To the left of the inflection point, the HR was 2.04 (95% CI: 1.69 to 2.46), while to the right, the HR was 0.89 (95% CI: 0.48 to 1.65).

**Conclusion:**

Our study reveals a non-linear relationship and a saturation effect between the TyG index and the development of prediabetes among young individuals in China, with an inflection point at 9.39. Understanding this non-linear relationship can assist clinicians in identifying young individuals at high risk and implementing targeted interventions to reduce their risk of progressing to diabetes.

## Introduction

Prediabetes is characterized by elevated blood glucose levels below the diagnostic threshold for diabetes but is associated with a higher risk of developing diabetes (1). The standardized prevalence of prediabetes in Chinese adults was 35.2% (33.5% to 37.0%) (2). Approximately 70% of prediabetes patients will progress to diabetes within 10 years, and the incidence of diabetes exceeds 90% within 20 years (3). Prediabetes is also associated with a high burden of cardiometabolic risk factors and poor outcomes (4). In a large meta-analysis of prospective studies (53 studies, 1.6 million individuals, median follow-up duration 9.5 years) examining the risks of cardiovascular disease and death in persons with prediabetes compared with normal glycemia (5), prediabetes was associated with an increased risk of cardiovascular disease and all-cause mortality. Several cohort studies have shown that individuals with prediabetes have a higher all-cause mortality rate compared to those with normal glycemia (6–12). Similarly, individuals with prediabetes have a high risk of hospitalization (11). Age is a significant risk factor for prediabetes, with evidence showing a strong association between the increase in prediabetes and age. In a 2011–2012 National Health and Nutrition Examination Survey (NHANES) analysis, in adults, the prevalence of prediabetes was 38.0%, while among young individuals aged 20–44 years, it reached up to 28.2% (13). Prediabetes is commonly considered a warning sign; nevertheless, many prediabetic patients, particularly younger individuals, tend to neglect this metabolic abnormality and underestimate its importance.

The triglyceride-glucose (TyG) index is calculated as the product of fasting plasma glucose (FPG) levels and triglyceride (TG) (14). Due to its convenience and ease of calculation, the TyG index has been widely used in various clinical settings (15–20). Insulin resistance (IR) is a key factor in metabolic disorders such as metabolic syndrome, non-alcoholic fatty liver disease (NAFLD), diabetes, and obesity (21, 22). IR is a major contributor to the progression from normal glucose resistance to prediabetes and then to diabetes. The TyG index is considered a novel marker of IR that accurately and reliably reflects the extent of IR (23, 24). Evidence suggests an association between the TyG index and the prevalence of prediabetes in middle-aged and older adults, but it is unclear whether this association exists in young adults (25).

Due to economic growth, lifestyle, and diet changes, diabetes rates are rising, with younger people increasingly affected. Young diabetics may show unusual symptoms and be overlooked, making early risk identification and intervention critical (26, 27). This study used existing data to examine the TyG index and prediabetes risk in individuals aged 20–45, aiming to support its clinical use for early prediabetes detection.

## Methods

### Study design

This study utilized data from a previous retrospective cohort study conducted by Chinese researchers (Chen et al.) (28). The target independent variable was the TyG at baseline. The outcome variable was the development from normoglycemia to prediabetes at follow-up.

### Data source

Access to the original dataset was granted at no cost through the DATADRYAD platform (www.datadryad.org), courtesy of Ying Chen et al. In accordance with Dryad’s usage policy, the data is available for academic and research purposes, allowing users to share, adapt, alter, and build upon the material, provided it is not for commercial use and proper attribution is given to the original authors and source. The dataset was sourced from a publicly accessible study published in 2018 titled “Association of body mass index and age with diabetes onset in Chinese adults: a population-based cohort study,” which can be found at http://dx.doi.org/10.1136/bmjopen-2018–021768. For those interested, the dataset can be retrieved from the following link: https://doi.org/10.5061/dryad.ft8750v (28). Given that the current study involves a secondary analysis of existing data, there was no need for obtaining informed consent or additional ethical approval.

### Research population

The primary research included 685,277 healthy individuals aged 20 years and above, all of whom had undergone a minimum of two health assessments. The study focused on participants who, during follow-up, had FPG levels ranging from 110 to 125 mg/dL without any prior diagnosis of diabetes. The initial selection excluded participants based on several factors (1): lack of detailed information regarding weight, height, or gender (2); BMI values outside the normal range (<15 kg/m^2^ or >55 kg/m^2^) (3); intervals between visits shorter than 2 years (4); missing fasting plasma glucose readings (5); individuals diagnosed with diabetes at the start or with uncertain diabetes status at the time of follow-up. Following these criteria, the study retained 211,833 participants.

**Figure 1:**
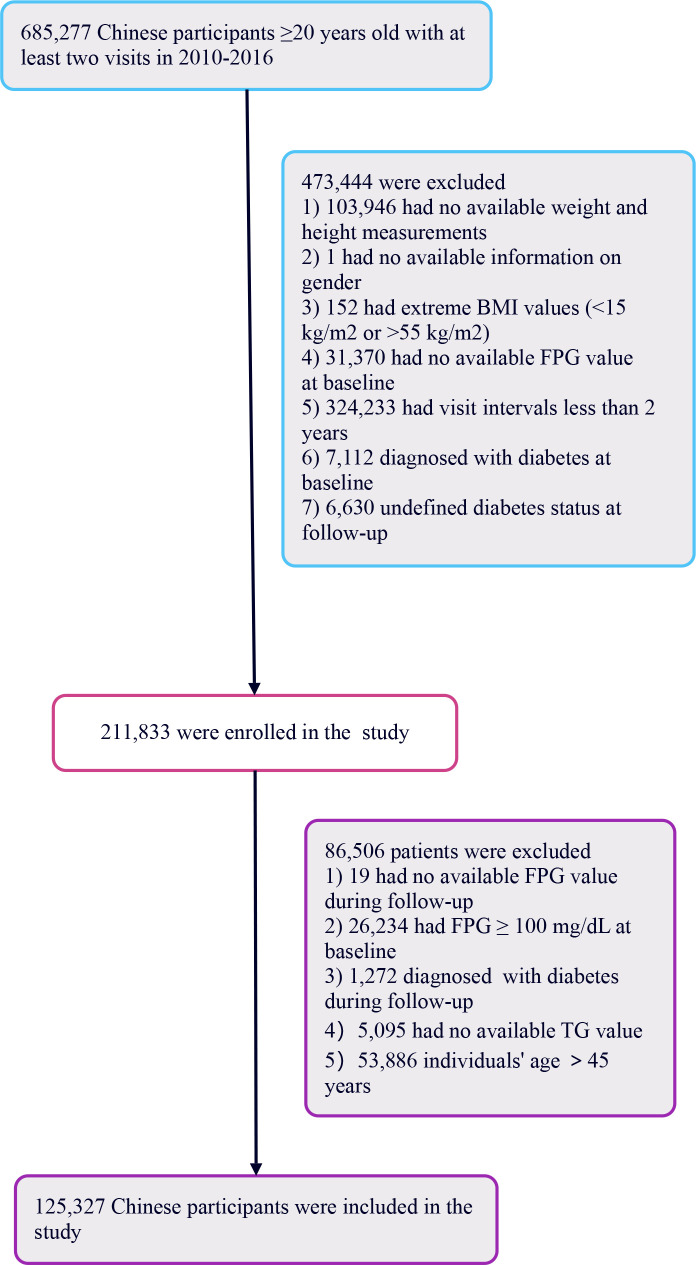
Flowchart illustrating the selection process of study participants.

Further analysis led to the exclusion of an additional 86,506 participants for reasons including: 1) absence of follow-up FPG readings, 2) baseline FPG levels ≥100 mg/dL, 3) diabetes diagnosis at follow-up, 4) had no available TG value, and 5) lack of TG values or being over 45 years of age. Young people are defined as those aged 45 years old or younger (29). Ultimately, the study included 125,327 healthy participants. The process of selecting participants for this study is depicted in **Figure 1**.

### Data collection

In this study, data collection included demographic information such as age, systolic blood pressure (SBP), diastolic blood pressure (DBP), height, and weight, from which body mass index (BMI) was calculated. To ensure consistency in data collection, staff received specialized training focusing on demographic data and key measurements, including blood pressure. Tests were uniformly conducted in a standardized laboratory environment for FPG, serum creatinine (Scr), TG, total cholesterol (TC), blood urea nitrogen (BUN), alanine aminotransferase (ALT), low-density lipoprotein cholesterol (LDL-c), and high-density lipoprotein cholesterol (HDL-c). Additionally, the study collected information on the patients’ smoking and drinking histories, defining current drinking as 1, former drinking as 2, never drinking as 3. Similarly, current smoking was coded as 1, former smoking as 2, never smoking as 3.

### Outcome measures

Our primary outcome was the occurrence of prediabetes, defined by FPG levels in the range of 100–125 mg/dL at follow-up without reported incident diabetes (30).

### Statistical analysis

The TyG index, the primary exposure variable in this study, is defined as follows: TyG index = ln [FPG (mg/dL) × TG (mg/dL)/2] (31). We divided it into four quartiles and considered it as a continuous variable. For continuous variables following a normal distribution, we reported the mean and standard deviation; for non-normally distributed data, we provided the median. For categorical variables, we presented the frequency and proportion. To compare differences between TyG index groups, we used the Kruskal-Wallis H test (for skewed distributed data), one-way analysis of variance (for normally distributed data), or chi-square test (for categorical variables).

We constructed several models to assess the relationship between the TyG index and prediabetes risk: a baseline model without any adjustments, a simplified model adjusting for gender and age only (Model I), and a comprehensive model adjusting for multiple covariates (Model II: including age, gender, BMI, systolic blood pressure, diastolic blood pressure, alanine aminotransferase, urea nitrogen, aspartate aminotransferase, total cholesterol, high-density lipoprotein cholesterol, low-density lipoprotein cholesterol, serum creatinine, family history of diabetes, alcohol consumption, and smoking status). We recorded the effect size (hazard ratio, HR) and its 95% confidence interval (CI) for each model.

We adjusted for potential confounding factors based on clinical experience, literature review, and univariate analysis results. Additionally, we used a multivariable Cox proportional hazards model and introduced cubic spline functions and smooth curve fitting to explore the possible nonlinear relationship between the TyG index and prediabetes risk. We also used a segmented Cox proportional hazards model to further clarify this nonlinear relationship. Unmeasured confounding between TyG and Prediabetes risk was assessed by calculating E-values.

To validate our findings, we conducted a series of sensitivity analyses. By incorporating continuous variables into a generalized additive model (GAM) in curve form, we further confirmed the robustness of the results. Additionally, we conducted analyses using stratified Cox proportional hazards models in different subgroups (such as age, gender, blood pressure, smoking, and drinking status). Finally, we used likelihood ratio tests to examine whether there were interactions in the model, both in models including interaction terms and those without. All analyses were performed using Empower Stats (X&Y Solutions, Inc., Boston, MA, http://www.empowerstats.com), with a statistical significance level set at a two-sided P value less than 0.05.

## Result

### The characteristics of participants

**Figure 2:**
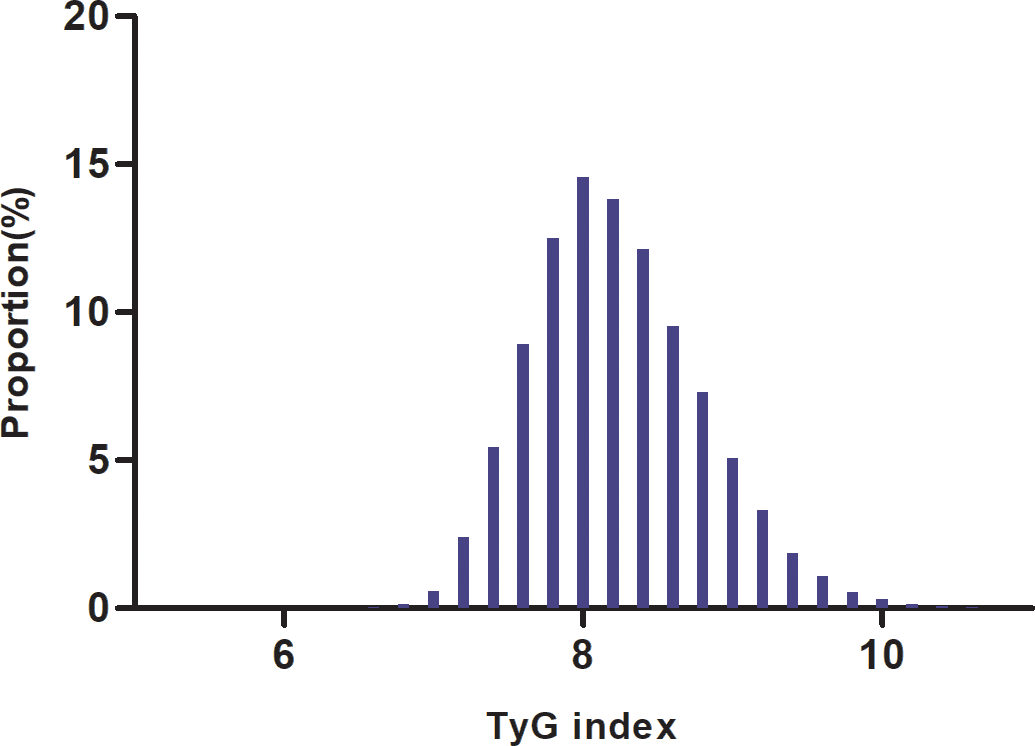
Distribution of TyG index. It presented a normal distribution, ranging from4.65 to 11.78, with a mean of 8.23.

We conducted a comprehensive analysis of the baseline characteristics of participants to investigate the relationship between the TyG index and the risk of prediabetes. The results are summarized in **Table 1**, demonstrating significant differences between normal and prediabetes groups across various parameters. The median follow-up duration of our study was 3.00 years, ranging from 2.00 to 6.20 years. The prediabetes group exhibited a slightly higher mean age (35.70 years) compared to the normal group (34.31 years), along with increased height, weight, and BMI. SBP and DBP were also notably elevated in the prediabetes group. The prediabetes group displayed higher levels of FPG, TG, and the TyG index, indicating a potential link between these parameters and the development of prediabetes. Additionally, AST, ALT, BUN, and Scr levels were higher in the prediabetes group. In addition, higher percentage of males, current smokers, and current drinkers were observed in the prediabetes group, along with a greater prevalence of family history of diabetes. Furthermore, the average follow-up duration was slightly longer in the prediabetes group compared to the normal group. As illustrated in **Figure 2**, a detailed Distribution of TyG index was conducted. It presented a normal distribution, ranging from 4.65 to 11.78, with a mean of 8.23.

**Table 1:** The baseline characteristics of participants.

TyG index	Normal	prediabetes	P-value
Participants	114,999	10,328	
Age (years)	34.31 ± 5.31	35.70 ± 5.32	<0.001
Height (cm)	166.98 ± 8.30	168.75 ± 8.13	<0.001
Weight (kg)	63.15 ± 12.39	68.90 ± 13.03	<0.001
BMI (kg/m^2^)	22.52 ± 3.27	24.08 ± 3.57	<0.001
SBP (mmHg)	114.74 ± 13.85	120.30 ± 14.59	<0.001
DBP (mmHg)	71.66 ± 9.77	75.11 ± 10.50	<0.001
FPG at baseline (mg/dL)	84.96 ± 8.59	90.10 ± 7.65	<0.001
TG (mg/dL)	102.15 ± 73.75	130.38 ± 98.60	<0.001
TyG index	8.21 ± 0.56	8.48 ± 0.61	<0.001
ALT (U/L)	16.50 (12.00–26.00)	22.00 (14.10–35.80)	<0.001
AST (U/L)	21.00 (18.00–25.20)	22.30 (19.00–28.00)	<0.001
BUN (mmol/L)	4.46 ± 1.11	4.65 ± 1.12	<0.001
Scr (μmol/L)	68.90 ± 15.10	72.65 ± 14.81	<0.001
TC (mmol/L)	4.52 ± 0.83	4.65 ± 0.85	<0.001
HDL-c (mmol/L)	1.38 ± 0.30	1.32 ± 0.29	<0.001
LDL-c (mmol/L)	2.63 ± 0.63	2.72 ± 0.63	<0.001
Sex			<0.001
Male	59,197 (51.48%)	7,039 (68.15%)	
Female	55,802 (48.52%)	3,289 (31.85%)	
Smoking status			<0.001
Current smoker	4,684 (14.19%)	620 (19.40%)	
Ever smoker	1,448 (4.39%)	169 (5.29%)	
Never	26,882 (81.43%)	2,407 (75.31%)	
Drinking status			<0.001
Current drinker	410 (1.24%)	60 (1.88%)	
Ever drinker	4,801 (14.54%)	602 (18.84%)	
Never	27,803 (84.22%)	2,534 (79.29%)	
Family history of diabetes			<0.001
No	112,693 (97.99%)	10,031 (97.12%)	
Yes	2306 (2.01%)	297 (2.88%)	
Follow-up (year)	3.12 ± 0.93	3.23 ± 0.96	<0.001

Continuous variables were summarized as mean (SD) or medians (quartile interval); categorical variables were displayed as percentage (%).

BMI, body mass index; SBP, systolic blood pressure; DBP; diastolic blood pressure; TG triglyceride; TC, total cholesterol; HDL-c, high-density lipoprotein cholesterol; LDL-c, low-density lipoprotein cholesterol; AST aspartate aminotransferase; ALT, alanine aminotransferase; BUN, blood urea nitrogen; Scr, serum creatinine; FPG, fasting plasma glucose; TyG index, triglyceride glucose index.

### Incidence of prediabetes in participants

**Figure 3:**
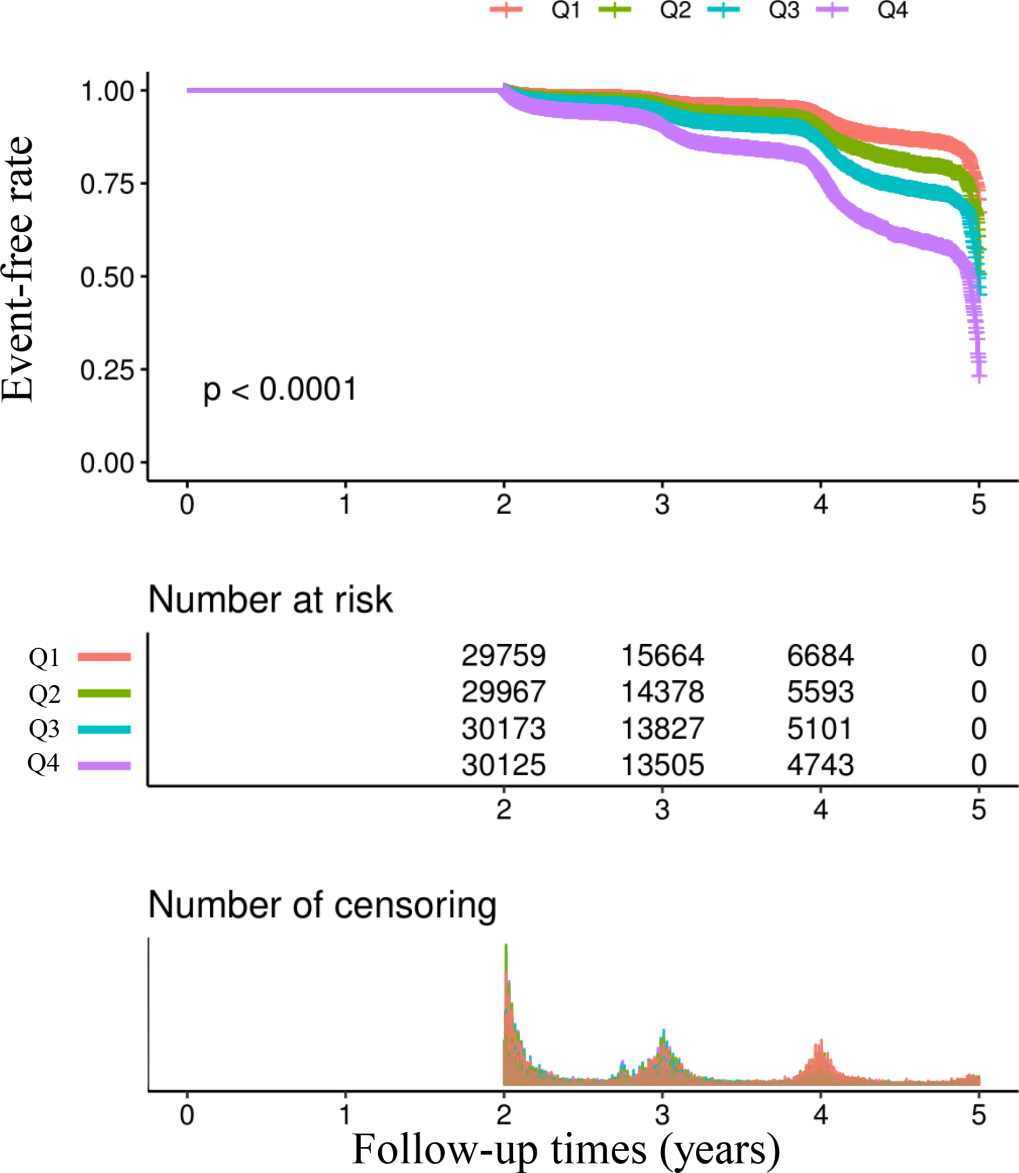
Kaplan–Meier event-free survival curve. Kaplan–Meier analysis of incident prediabetes based on two group (log-rank, P < 0.0001).

**Table 2** and **Figure 3** describe the incidence rates of prediabetes. Among the participants, 10,328 (8.24%) developed prediabetes. Participants were divided into subgroups based on the quartiles of the TyG index. The incidence rates of prediabetes per 1000 person-years were 14.426, 20.012, 27.066, and 44.490 for each TyG index quartile. The incidence rates of prediabetes in each TyG index quartile were as follows: Q1: 4.68%, Q2: 6.27%, Q3: 8.33%, and Q4: 13.69%. Participants with the highest TyG index (Q4) had a higher risk of developing prediabetes compared to those with the lowest TyG index (Q1) (trend p < 0.001).

**Table 2:** The Incidence rate of prediabetes (% or Per 1,000 person-year).

TyG index (quartile)	Participants (n)	Prediabetes events (n)	Incidence rate (95%CI) (%)	Per 1,000 person-year
Total	125327	10328	8.24 (8.09–8.39)	26.355
Q1	31,328	1466	4.68 (4.45–4.91)	14.426
Q2	31,317	1962	6.27 (6.00–6.53)	20.012
Q3	31,349	2611	8.33 (8.02–8.64)	27.066
Q4	31,332	4289	13.69 (13.31–14.07)	44.490
P for trend				<0.001

### Multivariable analysis using Cox proportional hazards regression model

In **Table 3**, the relationship between the TyG index and the risk of prediabetes is presented across different models. The results are as follows: In the crude model, the HR for the TyG index was 2.17 (95%CI 2.11–2.24, P < 0.0001). After adjusting for age and sex (Model I), the HR decreased to 1.94 (95%CI 1.88–2.00, P < 0.0001). Further adjustments in Model II, which included age, sex, SBP, DBP, BMI, ALT, AST, BUN, Scr, TC, LDL-C, HDL-c, family history of diabetes, drinking status, and smoking status, resulted in an HR of 1.81 (95%CI 1.54–2.13, P < 0.0001).

**Table 3:** Relationship between TyG index and risk of prediabetes in different models.

Exposure	Crude model (HR,95%CI) P	Model I (HR,95%CI) P	Model II (HR,95%CI) P	Model III (HR,95%CI) P
TyG index	2.17 (2.11, 2.24) <0.0001	1.94 (1.88, 2.00) <0.0001	1.81 (1.54, 2.13) <0.0001	1.86 (1.57, 2.19) <0.0001
(TyG index quartiles)				
Q1	Ref	Ref	Ref	Ref
Q2	1.49 (1.39, 1.59) <0.0001	1.40 (1.31, 1.50) <0.0001	1.29 (0.95, 1.75) 0.1065	1.30 (0.95, 1.77) 0.0998
Q3	2.11 (1.98, 2.25) <0.0001	1.86 (1.74, 1.99) <0.0001	1.75 (1.30, 2.34) 0.0002	1.81 (1.35, 2.44) <0.0001
Q4	3.56 (3.36, 3.78) <0.0001	2.90 (2.72, 3.09) <0.0001	2.33 (1.72, 3.16) <0.0001	2.44 (1.78, 3.34) <0.0001
P for trend	<0.0001	<0.0001	<0.0001	<0.0001

Crude model: we did not adjust other covariates.

Model I: we adjusted age, sex.

Model II: we adjusted age, sex, SBP, DBP, BMI, ALT, AST, BUN, Scr, TC, LDL-C, HDL-c, family history of diabetes, drinking status, and smoking status.

Model III: we adjusted age(smooth), sex, SBP (smooth), DBP (smooth), BMI (smooth), BUN (smooth), Scr (smooth), ALT (smooth), AST (smooth), TC (smooth), LDL-C(smooth), HDL-c(smooth), smoking status, drinking status, family history of diabetes.

HR, Hazard ratios; CI, confidence, Ref, reference.

After adjustments, similar trends were observed across all quartiles, indicating a significant association between the TyG index quartiles and the risk of prediabetes (P for trend < 0.0001 for all models).

### Sensitivity analysis

We performed a series of sensitivity analyses to confirm the reliability of our conclusions. Initially, the generalized additive model (GAM) in Model III, which included additional smooth terms for various variables, showed an HR of 1.86 (95%CI 1.57–2.19, P < 0.0001) (**Table 3**). Secondly, participants with a BMI ≥ 28 kg/m^2^ were excluded (32). After controlling for confounding variables, the results demonstrated a persistent positive association between the TyG index and the risk of prediabetes (HR= 1.82, 95% CI: 1.52–2.17, p < 0.0001). Furthermore, we performed a sensitivity analysis excluding patients aged ≥ 40 years. After adjusting for confounding variables, the results continued to demonstrate a positive association between the TyG index and the risk of prediabetes (HR = 1.99, 95% CI: 1.64–2.42, p < 0.0001). Additionally, we conducted an analysis on participants without a family history of diabetes, revealing a risk ratio of 1.83 (95% confidence interval: 1.54–2.16, p < 0.0001). Based on all sensitivity analyses, we concluded that our findings are reliable (**Table 4**).

**Table 4:** Relationship between TyG index and the risk of prediabetes in different sensitivity analyses.

Exposure	Model I (HR,95%CI) P	Model II (HR,95%CI) P	Model III (HR,95%CI) P
TyG index	1.82 (1.52, 2.17) <0.0001	1.99 (1.64, 2.42) <0.0001	1.83 (1.54, 2.16) <0.0001
(TyG index quartiles)			
Q1	Ref	Ref	Ref
Q2	1.33 (0.97, 1.82) 0.0792	1.18 (0.84, 1.65) 0.3502	1.21 (0.88, 1.67) 0.2386
Q3	1.77 (1.31, 2.40) 0.0002	1.59 (1.15, 2.20) 0.0053	1.67 (1.23, 2.26) 0.0010
Q4	2.39 (1.73, 3.29) <0.0001	2.14 (1.52, 3.01) <0.0001	2.25 (1.64, 3.09) <0.0001
P for trend	<0.0001	<0.0001	<0.0001

Model I was a sensitivity analysis performed after excluding participants with BMI≥ 28 kg/m^2^ (N= 8,595). we adjusted age, sex, BMI, SBP, DBP, ALT, AST, BUN, Scr, TC, LDL-C, HDL-c, family history of diabetes, drinking status, and smoking status.

Model II was a sensitivity analysis performed after excluding participants with age≥ 40 years (N= 25,255). we adjusted age, sex, BMI, SBP, DBP, ALT, AST, BUN, Scr, TC, LDL-C, HDL-c, family history of diabetes, drinking status, and smoking status.

Model III was a sensitivity analysis performed on participants without family of diabetes (N= 2,603). We adjusted age, sex, SBP, DBP, BMI, ALT, AST, BUN, Scr, TC, LDL-C, HDL-c, drinking status, and smoking status. HR, Hazard ratios; CI, confidence, Ref, reference.

### Results of the two-piece Cox proportional hazards regression model

**Figure 4:**
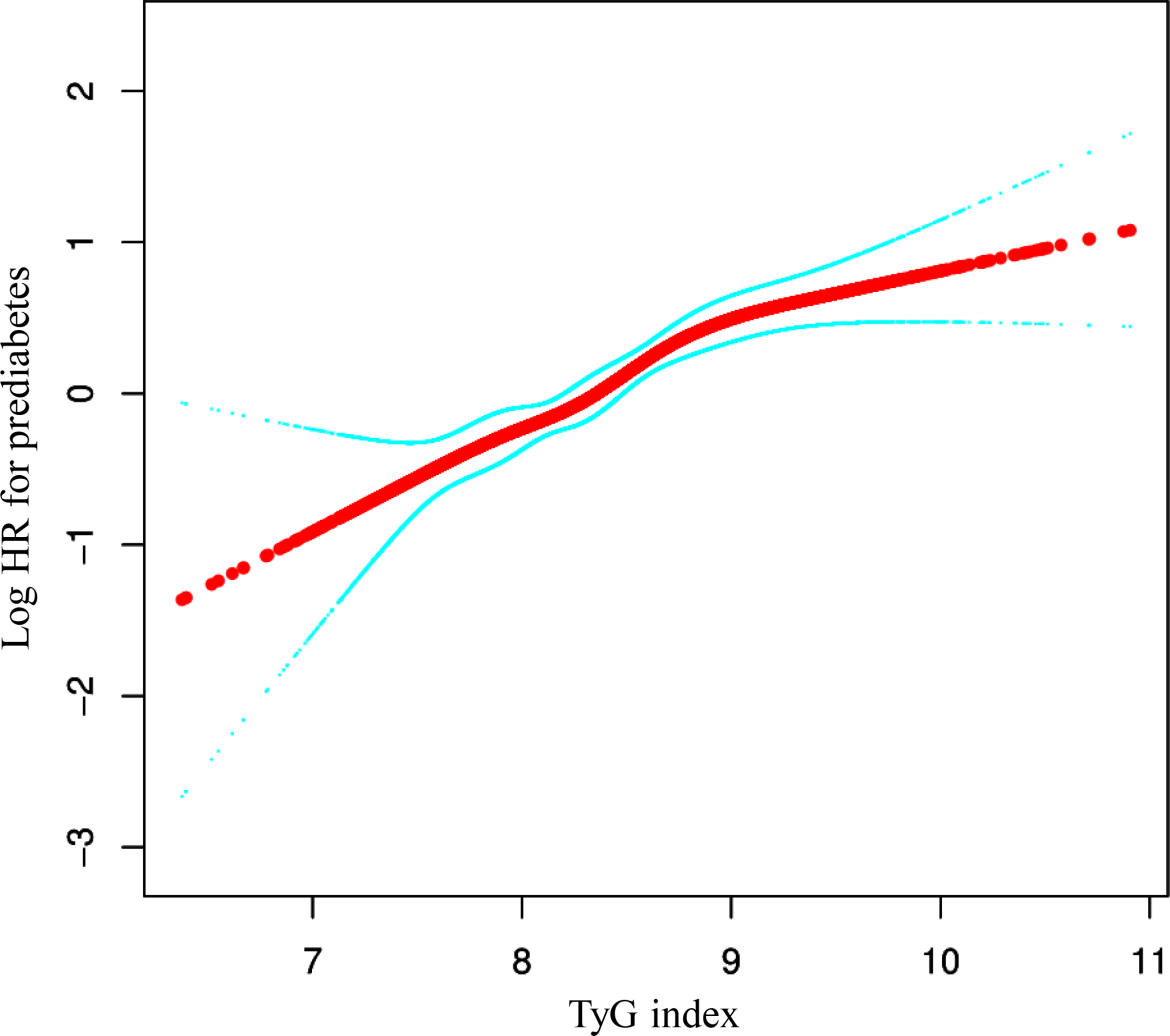
The non-linear relationship between TyG index and the risk of prediabetes in participants. We used a Cox proportional hazards regression model with cubic spline functions to evaluate the relationship between the TyG index and prediabetes risk. We adjusted age, sex, SBP, DBP, BMI, ALT, AST, BUN, Scr, TC, LDL-C, HDL-c, family history of diabetes, drinking status, and smoking status. The result showed that the relationship between the TyG index and prediabetes risk was non-linear, with the inflection point of TyG index being 9.39.

We identified a non-linear relationship between the TyG index and the risk of prediabetes (**Figure 4** and **Table 5**). Firstly, we used a Cox proportional hazards regression model with cubic spline functions to evaluate the relationship between the TyG index and prediabetes risk. The result showed that the relationship between the TyG index and prediabetes risk was non-linear. We employed a two-piecewise Cox proportional hazards regression model to investigate the relationship between the TyG index and the risk of developing prediabetes. The standard Cox regression model revealed a HR of 1.81 (95% CI: 1.54, 2.13) with a P value of <0.0001, indicating a significant association between the TyG index and prediabetes risk. In addition, we identified an inflection point in the TyG index at 9.39. Below this inflection point (<9.39), there is a significant positive correlation between the TyG index and the risk of prediabetes (HR: 2.04, 95% CI: 1.69, 2.46, P <0.0001). Conversely, Conversely, when the TyG index is 9.39 or higher, their relationship is not statistically significant (HR: 0.89, 95% CI: 0.48, 1.65, P=0.7019).

**Table 5:** The result of the two-piecewise Cox proportional hazards regression model.

Outcome: prediabetes	HR, 95% CI	P-value
Fitting model by standard Cox regression	1.81 (1.54, 2.13)	<0.0001
Fitting model by two-piecewise Cox regression		
Inflection points of TyG index	9.39	
<9.39	2.04 (1.69, 2.46)	<0.0001
≥9.39	0.89 (0.48, 1.65)	0.7019
P for log likelihood ratio test	0.011	

We adjusted age, sex, SBP, DBP, BMI, ALT, AST, BUN, Scr, TC, LDL-C, HDL-c, family history of diabetes, drinking status, and smoking status.

### Subgroup analysis results

As illustrated in **Table 6**, a detailed subgroup analysis was conducted. Gender, age, BMI, systolic and diastolic blood pressures, smoking and drinking habits, and a family history of diabetes did not alter the association between the TyG index and the risk of prediabetes. Thus, no significant interactions were observed between these variables and the TyG index (all interaction P > 0.05).

**Table 6:** Effect size of TyG index on prediabetes in prespecified and exploratory subgroups.

Variable	HR (95% CI)	P-value	P for interaction
**Age, years**			0.4260
20–30	2.14 (1.40, 3.25)	0.0004	
30–40	1.90 (1.57, 2.30)	<0.0001	
>40	1.66 (1.32, 2.08)	<0.0001	
**BMI (kg/m 2)**			0.7626
<18	2.73 (0.55, 13.53)	0.2186	
18–24	1.95 (1.56, 2.42)	<0.0001	
24–28	1.71 (1.38, 2.14)	<0.0001	
≥28	1.95 (1.39, 2.76)	0.0001	
**Sex**			0.1758
Male	1.74 (1.46, 2.06)	<0.0001	
Female	2.19 (1.59, 3.03)	<0.0001	
**SBP (mmHg)**			0.660
<140	1.79 (1.52, 2.12)	<0.0001	
≥140	2.00 (1.25, 3.20)	0.0040	
**DBP (mmHg)**			0.3484
<90	1.79 (1.52, 2.11)	<0.0001	
≥90	2.22 (1.42, 3.47)	0.0005	
**Drinking status**			0.3930
Current drinker	2.93 (1.20, 7.14)	0.0183	
Ever drinker	1.66 (1.30, 2.12)	<0.0001	
Never	1.87 (1.55, 2.24)	<0.0001	
**Smoking status**			0.2937
Current smoker	1.58 (1.22, 2.06)	0.0006	
Ever smoker	2.38 (1.43, 3.96)	0.0008	
Never	1.86 (1.55, 2.23)	<0.0001	
**Family history of diabetes**			0.2497
Yes	1.86 (1.57, 2.19)	<0.0001	
No	1.49 (1.03, 2.16)	0.0360	

Above model adjusted for age, sex, SBP, DBP, BMI, ALT, AST, BUN, Scr, TC, LDL-C, HDL-c, family history of diabetes, drinking status, and smoking status. In each case, the model is not adjusted for the stratification variable when the stratification variable was a categorical variable.

## Discussion

This retrospective cohort study revealed that a higher TyG index independently predicts an increased risk of prediabetes in Chinese adults aged 20–45. Furthermore, a nonlinear relationship was observed, indicating that the risk of prediabetes tends to rise with an increasing TyG index. Individuals in the highest TyG index quartile had a 2.33-fold increased risk of developing prediabetes compared to those in the lowest quartile. Additionally, a threshold effect curve was identified, revealing varying relationships between the TyG index and prediabetes risk across the inflection point.

Initially, the TyG index has been widely validated as an indicator of IR in various epidemiological studies. A cross-sectional study conducted in Mexico involving 99 individuals identified the TyG index as an optimal tool for assessing IR, showing a high sensitivity (96.5%) and good specificity (85.0%) compared to the gold standard, HIEC (23). Research involving 82 Brazilian subjects corroborated the TyG index as a more precise predictor of IR compared to HOMA-IR (33). Numerous studies have indicated an association between the TyG index and cardiovascular disease (CVD) risk (34–37). A study involving 4,340 American patients under the age of 65 with prediabetes or diabetes found that an increase in the TyG index is associated with a higher incidence of CVD, including congestive heart failure (CHF), coronary heart disease (CHD), atherosclerotic cardiovascular disease (ASCVD), heart attack, angina, and stroke (34). Additionally, several studies have found a close relationship between the TyG index and coronary artery disease (35–37). Furthermore, recent studies have identified a significant correlation between the TyG index and both cognitive and physical impairments in elderly individuals with prefrail hypertension (38).

Recently, there has been a growing body of research exploring the relationship between the TyG index and the risk of diabetes (39–42). A prospective cohort study conducted in rural China suggests that an increasing TyG index is associated with a higher cumulative risk of developing incident T2DM among individuals with normal weight (43). Another retrospective cohort study demonstrated a link between elevated TyG index levels and an augmented risk of diabetes within the Chinese population (44). Additionally, recent studies have indicated that there is a positive correlation between the TyG index and the risk of diabetes (45). It is noteworthy that prediabetes is a critical period for promoting, preventing, or delaying the development of diabetes mellitus (DM).

But research on the TyG index’s association with prediabetes is limited. An initial study demonstrated that the TyG index is comparable to HbA1C as a diagnostic marker for prediabetes (46). Findings from a case-control study conducted in Palembang, Indonesia, involving 570 participants without a family history of T2DM (265 prediabetes cases and an equal number of age-matched controls), indicated that the TyG index was the primary risk predictor for prediabetes (47). A prospective cohort study in China indicated that the predictive capacity of the TyG index in forecasting prediabetes surpassed that of obesity, lipid profiles, and other non-insulin-based IR indices (48). A cross-sectional study conducted in the United States, involving 25,159 participants, found that 23.88% had prediabetes and 16.22% were diagnosed with diabetes. The study revealed a positive relationship between the TyG index and the prevalence of prediabetes and diabetes (49). Interestingly, a cohort study conducted in China indicated that, among prediabetic patients, an elevated TyG-BMI might reduce the likelihood of returning to normal glucose levels in the future (50). However, no specific studies have investigated the association between TyG index and prediabetes in a young population.

Therefore, we hypothesize that the TyG index is positively correlated with the risk of prediabetes in young populations. Therefore, our study investigates the TyG index’s role in identifying prediabetes risk factors among young individuals, aiming to enhance prevention strategies for diabetes onset and progression within this demographic. Initially, our findings indicate the TyG index as a reliable predictor of prediabetes and an independent risk factor, consistent with existing research [45–46]. Furthermore, our study has shown a positive correlation between the TyG index and prediabetes, consistent with our research hypothesis. That is for every 1-unit increase in the TyG index, the risk of prediabetes will increase by 81% (HR: 1.81, 95% CI 1.54–2.13, P < 0.0001). Similarly, a cohort study conducted in China also identified a positive correlation between TyG and the risk of prediabetes after adjusting for variables (48). However, there are significant differences between the two studies in terms of subjects, outcomes, and adjusted variables. Their study sample is relatively small compared to ours. They also adjusted for different variables, such as age, cigarette smoking, alcohol consumption, education level, family history of diabetes, hypertension, and cardiovascular disease history, which differs slightly from our study. Additionally, they did not use the Cox proportional hazards regression model combined with cubic splines and smoothing curve fits to examine the nonlinear association between the TyG index and the risk of prediabetes. A study in the United States also found a nonlinear relationship between the TyG index and the prevalence of prediabetes and diabetes, identifying an inflection point at 8 (49), which is very similar to our conclusion but with some notable differences. Firstly, their study was cross-sectional, which has limitations such as a smaller sample size, unclear causality, and selection bias. Secondly, there are differences in the age distribution of the study populations between the two studies. Additionally, variations in the adjusted variables between the two studies may impact the research outcomes. Although both studies confirm a nonlinear relationship, we identified a critical threshold of 9.39. Our study focused on prediabetes as the outcome, while their study included both prediabetes and diabetes, which could be the primary reason for the earlier inflection point in their findings.

Our study has significant clinical implications. We have identified a non-linear relationship with a saturation effect between the TyG index and the risk of prediabetes in young individuals. Monitoring TyG index values can facilitate early interventions in young individuals to decrease the occurrence of prediabetes. Specifically, when the TyG index is below 9.39, by lowering FBG and TG, we can significantly reduce the risk of prediabetes. However, when the TyG index exceeds 9.39, merely controlling FBG and TG is insufficient to reduce the risk of prediabetes. Thus, it is necessary to manage other related risk factors such as BMI, smoking, and alcohol consumption to better prevent the onset of the disease. Consequently, it is advisable for young people to start making lifestyle changes early, including reducing the intake of high-fat foods, increasing physical activity, and controlling blood sugar levels. These measures can effectively help reduce the risk of developing prediabetes.

This study has some advantages. Firstly, we first identified a nonlinear relationship between the TyG index and prediabetes risk in young individuals. Furthermore, this study included a large sample of 125,327 young adults, minimizing potential biases. Sensitivity analysis was conducted to ensure the reliability and robustness of the results, and a two-stage Cox proportional hazards regression model was applied to determine the inflection point of the TyG index. These findings underscore the critical threshold between the TyG index and diabetes risk, emphasizing the importance of TyG index monitoring and early intervention strategies in diabetes prevention efforts. Additionally, subgroup analysis and interaction tests were performed. The results revealed variations in the impact of the TyG index on prediabetes risk across different subgroups, yet interaction analysis did not indicate significant interactions between the TyG index and factors such as drinking status, smoking status, or family history of diabetes. This further validates the stability of the results.

This study has several limitations. Firstly, as all participants were of Chinese descent, further research is needed to determine the relationship between the TyG index and the risk of prediabetes in individuals with different genetic backgrounds. Secondly, since this large-scale cohort study only focused on young adults in China, these findings may not be applicable to other racial groups and specific populations, such as children and the elderly. Thirdly, the average follow-up time for participants was relatively short. Future research could benefit from extending follow-up duration to mitigate potential misinterpretations or chance findings from short-term observations. Fourthly, there was no data available on serum insulin levels to compare the predictive value of the TyG index and HOMA-IR; additionally, there was also no data on the use of lipid-lowering medications, which could potentially impact the results. Fively, this report represents a secondary analysis of existing databases. Although adjustments were made for many confounding factors, variables not included in the database were not adjusted for. However, we calculated the E value to quantify the potential impact of unmeasured confounders and found that unmeasured confounders were unlikely to explain the results. sixly, The current diagnostic criteria for prediabetes have some limitations, primarily relying on impaired fasting glucose and not considering oral glucose tolerance tests, glycated hemoglobin, and multiple fasting glucose measurements. This single-index approach may lead to underdiagnosis of prediabetes. Therefore, future studies evaluating the incidence of prediabetes should consider measuring additional variables, including oral glucose tolerance tests, glycated hemoglobin, and multiple fasting glucose measurements, to improve diagnostic accuracy and comprehensiveness. Additionally, this retrospective observational study provides an associative inference rather than establishing a causal relationship between the TyG index and the risk of prediabetes. Lastly, this study only measured the TyG index and other parameters at baseline and did not consider changes in the TyG index over time. Therefore, in the future, we should collect as much information as possible, including information on changes in the TyG index.

## Conclusion

This study reveals a nonlinear relationship and a saturation effect between the TyG index and the risk of prediabetes among individuals aged 20 to 45 in China. There is a significant positive correlation between the TyG index and the risk of prediabetes to the left of the inflection point at a TyG index of 9.39. Reducing FBG and TG levels can significantly decrease the risk of prediabetes. Conversely, when the TyG index reaches or exceeds 9.39, its relationship with the risk of prediabetes is no longer statistically significant; merely lowering the TyG index is not enough to reduce the risk of prediabetes. It is also necessary to comprehensively control other factors such as BMI, smoking, and alcohol consumption. Understanding this non-linear relationship can help clinicians identify high-risk young individuals and implement focused interventions to reduce the risk of developing diabetes.

## Data Availability

Publicly available datasets were analyzed in this study. This data can be found here: https://bmjopen.bmj.com/content/8/9/e021768.
